# Robotic-assisted versus laparoscopic colorectal surgery: a meta-analysis of four randomized controlled trials

**DOI:** 10.1186/1477-7819-12-122

**Published:** 2014-04-26

**Authors:** Guixiang Liao, Zhihong Zhao, Shuhui Lin, Rong Li, Yawei Yuan, Shasha Du, Jiarong Chen, Haijun Deng

**Affiliations:** 1 Department of Radiation Oncology, Nanfang Hospital, Southern Medical University, No. 1838, Guangzhou Avenue North, Guangzhou 510515, China; 2 Institute of Nephrology and Urology, Third Affiliated Hospital of Southern Medical University, Guangzhou 510630, China; 3 Department of General Surgery, Nanfang Hospital, Southern Medical University, No. 1838, Guangzhou Avenue North, Guangzhou 510515, China

**Keywords:** Colorectal, Robotic, Laparoscopic, Meta-analysis, Colorectal cancer

## Abstract

**Background:**

Robotic-assisted laparoscopy is popularly performed for colorectal disease. The objective of this meta-analysis was to compare the safety and efficacy of robotic-assisted colorectal surgery (RCS) and laparoscopic colorectal surgery (LCS) for colorectal disease based on randomized controlled trial studies.

**Methods:**

Literature searches of electronic databases (Pubmed, Web of Science, and Cochrane Library) were performed to identify randomized controlled trial studies that compared the clinical or oncologic outcomes of RCS and LCS. This meta-analysis was performed using the Review Manager (RevMan) software (version 5.2) that is provided by the Cochrane Collaboration. The data used were mean differences and odds ratios for continuous and dichotomous variables, respectively. Fixed-effects or random-effects models were adopted according to heterogeneity.

**Results:**

Four randomized controlled trial studies were identified for this meta-analysis. In total, 110 patients underwent RCS, and 116 patients underwent LCS. The results revealed that estimated blood losses (EBLs), conversion rates and times to the recovery of bowel function were significantly reduced following RCS compared with LCS. There were no significant differences in complication rates, lengths of hospital stays, proximal margins, distal margins or harvested lymph nodes between the two techniques.

**Conclusions:**

RCS is a promising technique and is a safe and effective alternative to LCS for colorectal surgery. The advantages of RCS include reduced EBLs, lower conversion rates and shorter times to the recovery of bowel function. Further studies are required to define the financial effects of RCS and the effects of RCS on long-term oncologic outcomes.

## Background

Minimally invasive surgery for colorectal disease was introduced in 1991 [1]. Such surgery has been widely used worldwide and has become increasingly popular. However, technical barriers, including unstable video camera imaging, loss of dexterity and a steep learning curve, preclude the widespread adoption of minimally invasive surgery techniques for colorectal disease. To overcome these technical drawbacks, robotic surgical systems were introduced. Robotic laparoscopic colorectal surgery (RCS) was first reported in 2002 [2]. Since then, many studies of RCS have been widely reported [3-5]. RCS has some advantages over conventional laparoscopic surgery (LCS). These advantages include a three-dimensional image, convenient movements of the robotic arm, no tremor, motion scaling, a short learning curve, dexterity and ambidextrous capability [4,6,7]. Some studies have already proven that robotic laparoscopic colorectal surgery is associated with certain benefits compared to conventional laparoscopic colorectal surgery [8-10]. Additionally, the learning curve for robotic surgery is short [11]. However, the relative merits of RCS versus LCS are controversial. The aim of this study was to compare the efficacy and safety of RCS and LCS via a meta-analysis of the available randomized controlled trials (RCTs).

## Methods

### Research method

Two authors (XGL, HZZ) participated in this search to independently identify all published RCT studies comparing RCS and LCS. The Pubmed, Web of Science and Cochrane Library databases were searched. The following medical subject heading terms and key words were used: robotic; telerobotic; colorectal; colon; rectal; random. None of the searches had any language limitations, and the most recent search time was 1 March 2013.

### Inclusion criteria

For inclusion in this meta-analysis, the studies were required to meet the following criteria: (1) RCTs; (2) studies comparing the efficacy and safety of RCS and LCS for the treatment of colorectal disease; (3) studies that effectively reported both primary results and data adequate for meta-analysis.

### Data extraction

The effective data from all eligible RCTs were extracted by two reviewers (XGL, HZZ). The extracted data included the following: first author; year and country of publication; surgical technique; operation type; basic patient characteristics, including age, body mass index (BMI), number of patients, diagnosis, tumor stage, intraoperative data, postoperative data; and pathological details including operation time, estimated blood loss, open conversion rate, time to recovery of bowel function, length of hospital stay, complication rate, proximal margin, distal margin, harvested lymph nodes and cost. All available data were extracted from the relevant texts, tables and figures. In cases of disagreement about whether a study was suitable for inclusion in the meta-analysis, an additional reviewer (WYY) assessed the article and made the final decision.

### Risk of bias

Two reviewers (XGL, HZZ) independently evaluated the bias of each study using the Cochrane tools [12]. The assessment item included sequence generation, allocation of sequence concealment, blinding of participants and personnel, blinding of outcomes and assessments, incomplete outcome data, selective outcome reporting and other biases. When disagreement existed, the third reviewer (WYY) discussed the issues with the group and made a final conclusion.

### Statistical analyses

This meta-analysis was performed using the Review Manager (RevMan) software, version 5.2, provided by the Cochrane Collaboration. Dichotomous variables were analyzed using estimations of the odds ratio (OR) and 95% CI, and continuous variables were analyzed using weighted mean difference (WMD) and 95% CI. Fixed-effects models or random-effects models were applied. Heterogeneity was evaluated using χ^2^ and *I*^2^ tests. We considered heterogeneity to be present when the *I*^2^ statistic was >50%, and random-effects models were adopted in these cases. However, when the *I*^2^ statistic was <50%, we used fixed-effects models. *P* <0.05 was considered significant.

## Results

### Literature search results

**Figure 1:**
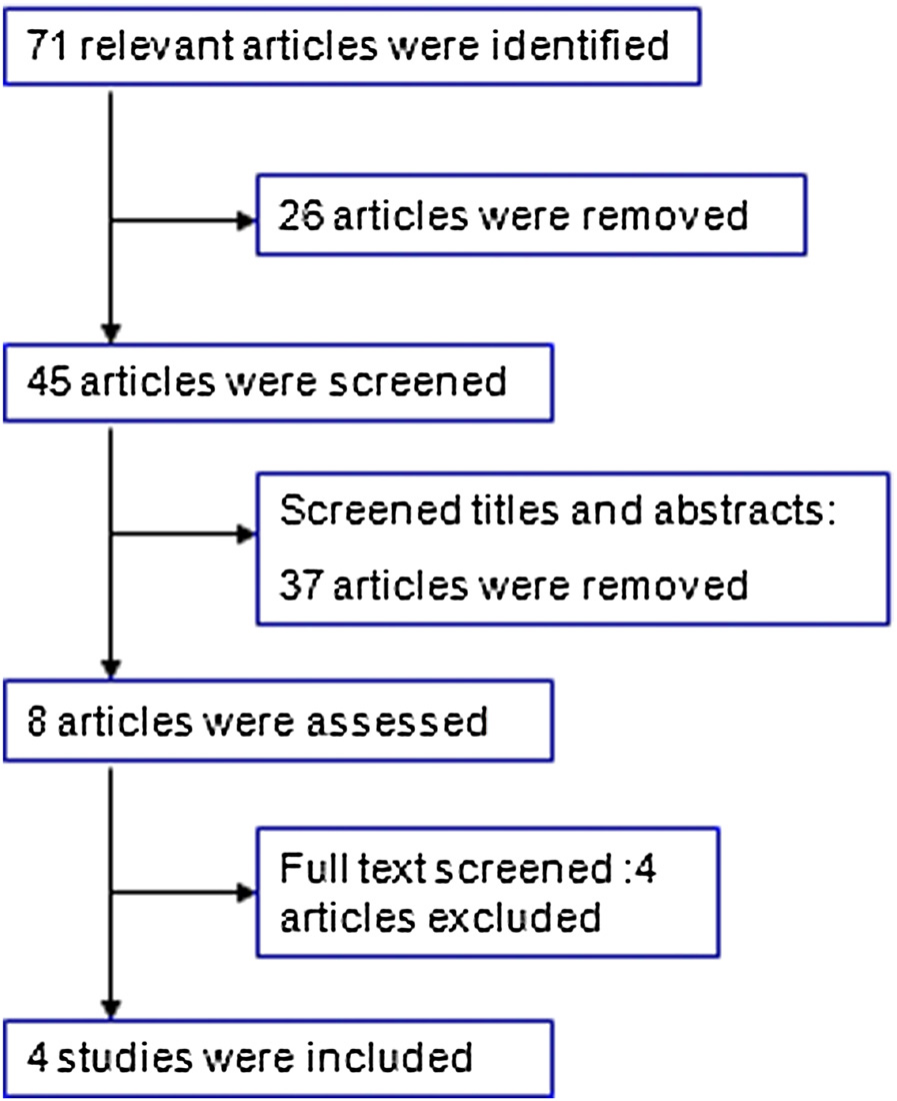
Flow chart of the literature search.

A total of 71 potentially relevant articles were identified, and the selection process is illustrated in Figure 1. After examination of the titles, abstracts and full texts, four published RCT studies [13-16] were found to meet all of the inclusion criteria and were entered into this meta-analysis. In total, 110 patients underwent RCS, and 116 patients underwent LCS. One study of right-sided colonic carcinoma [13], two studies of rectal cancer [14,15], and another study that included sigmoid colon cancer and rectal cancer [16] were reported. Information about the general characteristics of the studies and the participants is listed in Table 1, and the extracted data are shown in Table 2.

**Table 1:** General characteristics of the studies and participants

**Author**	**Year**	**Country**	**Group**	**N**	**Sex**	**BMI**	**ASA grade**	**Age, years**	**Disease**	**Tumor stage**	**Operation type**
					**M/F**		**I/II/III/IV**			**I/ II/III/IV**	
Baik SH	2008	Korea	RCS	18	14/4	22.8 (1.8)	12/6/0/0	57.3 (6.3)	Rectal cancer	5/4/9/0	Tumor-specific mesorectal excision
			LCS	18	14/4	24.0 (2.5)	9/6/1/1	62.0 (9.0)		5/4/9/0	
Jimenez RR	2011	Spain	RCS	28	12/16	28.59 (2.5)	14/14^a^	68.0 (9.1)	Sigmoid colon cancer and rectal cancer	15/13^b^	Total or subtotal mesorectal excision
			LC S	28	17/11	26.75 (5.6)	20/8^a^	61.5 (15.0)		21/7^b^	
Park JS	2012	Korea	RCS	35	14/21	24.4 (2.5)	15/16/4/0	62.8 (10.5)	Right-sided colon carcinoma	10/16/9/0	Right colectomies
			LCS	35	16/19	23.8 (2.7)	21/12/2/0	66.5 (11.4)		9/16/10/0	
Patriti A	2009	Italy	RCS	29	11/18	24 (6.2)	2/13/14/0	68 (10)	Rectal cancer	11/9/7/2	Total mesorectal excision
			LCS	37	12/25	25.4 (6.44)	2/14/21/0	69 (10)		17/8/10/2	

Values are expressed as mean (SD) or number. BMI, body mass index; ASA, American Society of Anesthesiologists; RCS, robotic-assisted colorectal surgery; LCS, laparoscopic colorectal surgery; M/F, male/female. ^a^(I + II)/ III; ^b^(T1 + T2)/T3.

**Table 2:** Data extracted from the included studies

**Study**	**Group**	**N**	**Operation time, minutes**	**EBL, ml**	**Open conversion events, n**	**Time to recovery of bowel function, d**	**Complication events, n**	**LOS, d**	**Proximal margin, cm**	**Distal margin, cm**	**Harvested lymph nodes, n**	**Total hospital cost, $**
Baik SH	RCS	18	217.1 (51.6)	N/A	0	1.8 (0.4)	4	6.9 (1.3)	10.9 (1.7)	4 (1.1)	20.1 (9.1)	N/A
	LCS	18	204.3 (56.9)		2	2.4 (1.3)	1	8.7 (1.7)	10.3 (3.6)	3.7 (1.1)	17.4 (10.6)	
Jimenez RR	RCS	28	159.4 (43.5)	N/A	2	N/A	4	N/A	N/A	4.8 (1.6)	17.6 (9.2)	N/A
	LCS	28	135.1 (29.2)		2		4			3.8 (0.7)	14.9 (8.7)	
Park JS	RCS	35	195 (41)	35.8 (26.3)	0	2.6 (1.4)	6	7.9 (4.1)	18.6 (7.3)	18 (9)	29.9 (14.7)	12,235 (1,907.9)
	LCS	35	130 (43)	56.8 (31.3)	0	2.9 (2.2)	7	8.3 (4.2)	18.3 (9.9)	14.5 (8)	30.8 (13.3)	10,320.7 (1,607.7)
Patriti A	RCS	29	202 (12)	137.4 (156)	0	N/A	9	11.9 (7. 5)	N/A	2.1 (0.9)	10.3 (4)	N/A
	LCS	37	208 (7)	127 (169)	7		7	9.6 (6.9)		4.5 (7.2)	11.2 (5)	

Values are expressed as mean (SD) or number. EBL, estimated blood loss; LOS, length of hospital stay; N/A, not applicable; RCS, robotic-assisted colorectal surgery; LCS, laparoscopic colorectal surgery.

### Risk of bias assessment

**Figure 2:**
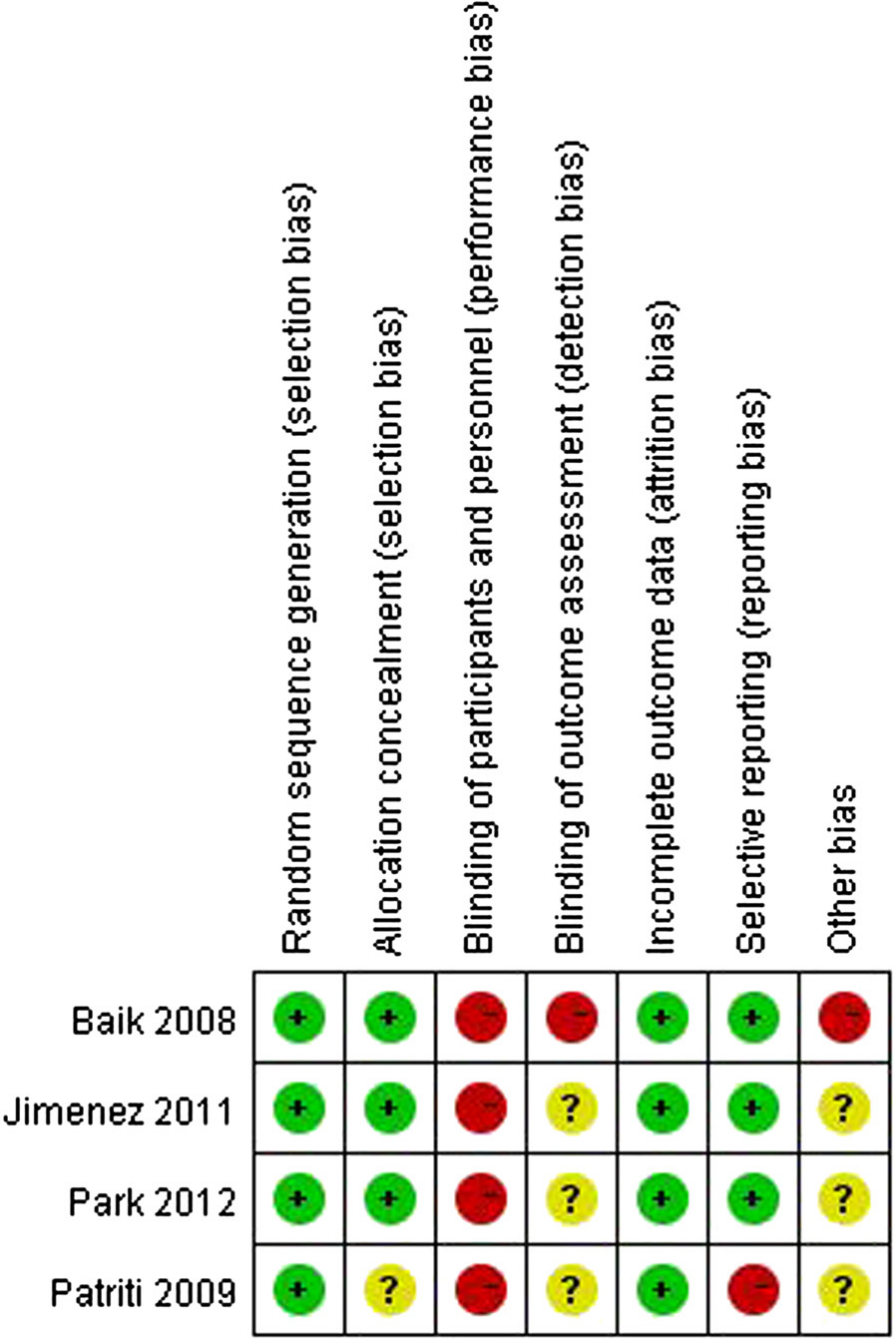
**Risk of bias graph.** (+), low risk of bias; (-), high risk of bias; (?), unclear risk of bias.

**Figure 3:**
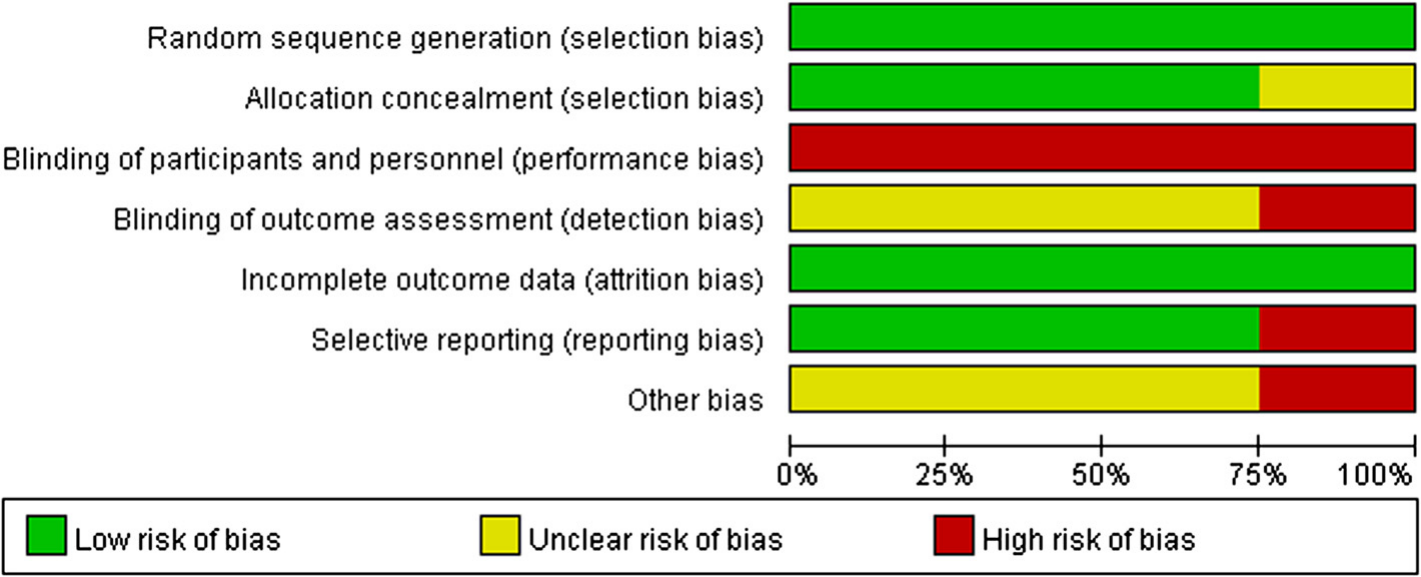
Risk of bias summary.

All of the studies were RCTs, but none were double- or even single-blinded. Assessments of the risk of bias are illustrated in Figure 2 and Figure 3.

### Meta-analysis

#### *Operation times*

**Figure 4:**
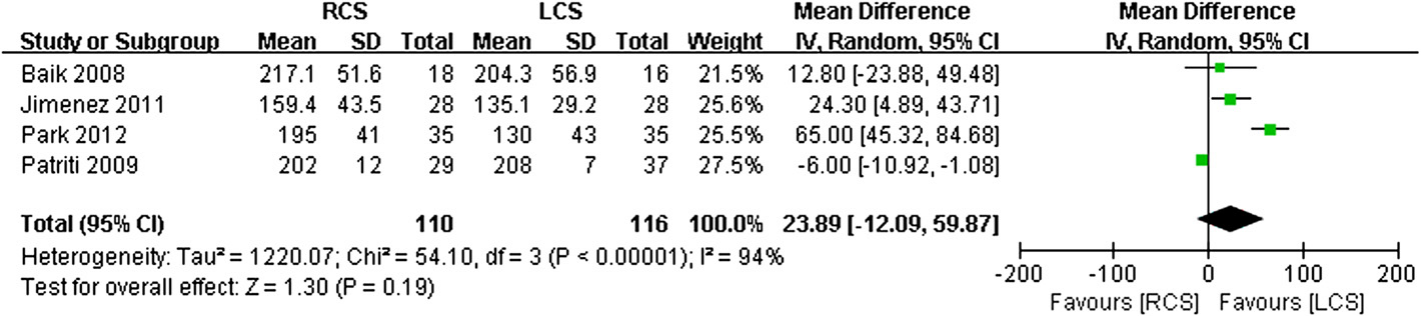
**Meta-analysis of the operation times shown as a forest plot between robotic-assisted colorectal surgery (RCS) and laparoscopic colorectal surgery (LCS).** The mean differences with 95% CIs are shown.

All four of the studies reported operation times. Two of the four studies indicated a tendency toward longer operation times for the RCS groups than for the LCS groups [13,16]. However, no significant differences between the two groups were reported in the other two studies [14,15]. The pooled data revealed that the operative times were not significantly different between the two techniques (WMD 23.89, 95% CI 12.09 to 59.87; *P* = 0.19), but the heterogeneity was high (*P* <0.001, *I*^2^ = 94%) (Figure 4).

### Estimated blood losses

**Figure 5:**
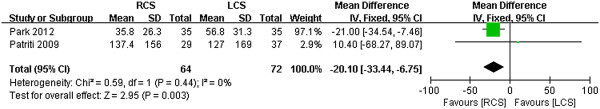
**Meta-analysis of the EBLs shown as a forest plot between robotic-assisted colorectal surgery (RCS) and laparoscopic colorectal surgery (LCS).** The mean differences with 95% CIs are shown.

Only two of the studies described intraoperative estimated blood losses (EBLs) [13,14]. Both of these studies reported that the EBLs did not significantly differ between the two approaches. In the studies of Baik *et al*., the hemoglobin change (g/dl) indices before and after the surgical inventions were reported. These numbers revealed no significant difference between the two techniques [15]. The pooled data from these studies suggested that the EBL of the RCS group was significantly lower than that of the LCS group (WMD -20.10, 95% CI -33.44 to -6.75; *P* = 0.003). Furthermore, there was no evidence of observed heterogeneity (*P* = 0.59, *I*^2^ = 0%) (Figure 5).

### Conversion rate

**Figure 6:**
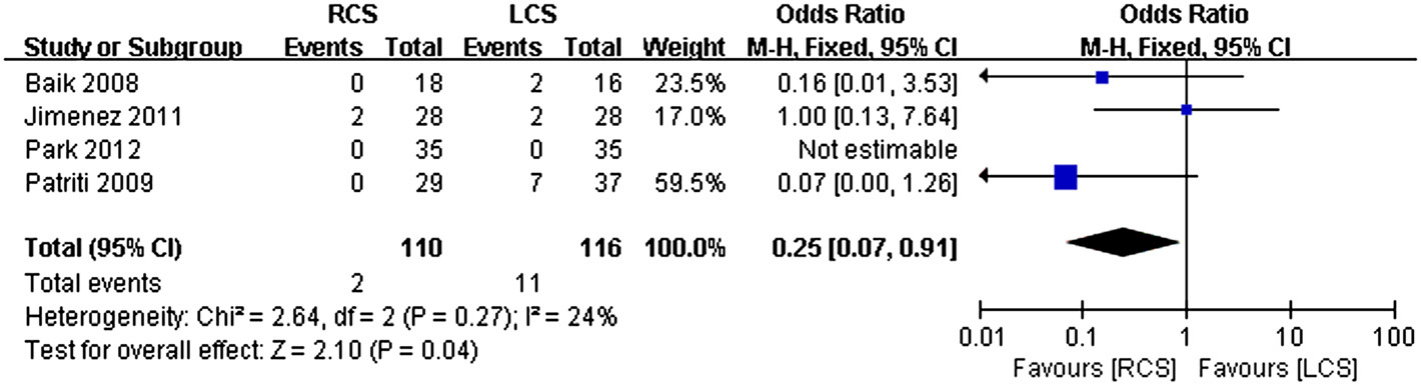
**Meta-analysis of the conversion rates shown as a forest plot between robotic-assisted colorectal surgery (RCS) and laparoscopic colorectal surgery (LCS).** The odds ratios with 95% CIs are shown.

Overall, conversions to open operations were required for two patients in the RCS group (1.82%) and for 11 patients in the LCS group (9.48%). Analyses of the pooled data revealed that the conversion rate was significantly lower in the RCS group than in the LCS group (WMD 0.25, 95% CI 0.07 to 0.91; *P* = 0.04), and there was no obvious heterogeneity among the four studies (*P* = 0.27, *I*^2^ = 24%) (Figure 6).

### Time to recovery of bowel function

**Figure 7:**
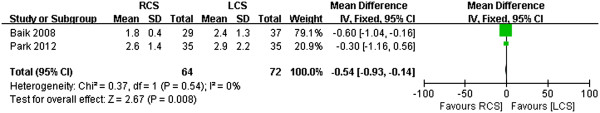
**Meta-analysis of the times to the recovery of bowel function shown as a forest plot between robotic-assisted colorectal surgery (RCS) and laparoscopic colorectal surgery (LCS).** The mean differences with 95% CIs are shown.

The time to first flatus was reported by Park *et al*. [13], and the number of days to peristalsis was reported by Baik *et al*. [15]; both of these variables are indicative of the time to the recovery of bowel function. Analyses of the pool data revealed that the RCS group exhibited shorter times to the recovery of bowel function than did the LCS group (WMD 0.54, 95% CI -0.93 to -0.14; *P* =0.008, *I*^2^ = 0) (Figure 7).

### Length of hospital stay

**Figure 8:**
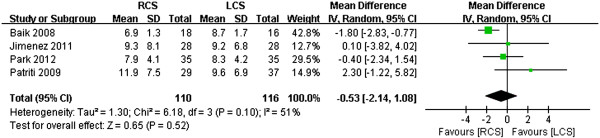
**Meta-analysis of length of hospital stays shown as a forest plot between robotic-assisted colorectal surgery (RCS) and laparoscopic colorectal surgery (LCS).** The mean differences with 95% CIs are shown.

Four of the studies reported the duration of hospital stays. Baik *et al*. [15] revealed that the mean length of the hospital stay (LOS) for all patients who underwent laparoscopic procedures was longer than that of those who underwent robotic procedures (*P* <0.001). However, the other three studies reported no differences in hospital stays between the two groups. Analysis of all of the studies revealed no significant difference between the two techniques in terms of LOS (WMD -0.53, 95% CI -2.14 to 2.08; *P* = 0.52). However, the heterogeneity was slightly high (*P* = 0.19, *I*^2^ = 51%) (Figure 8).

### Complication rate

**Figure 9:**
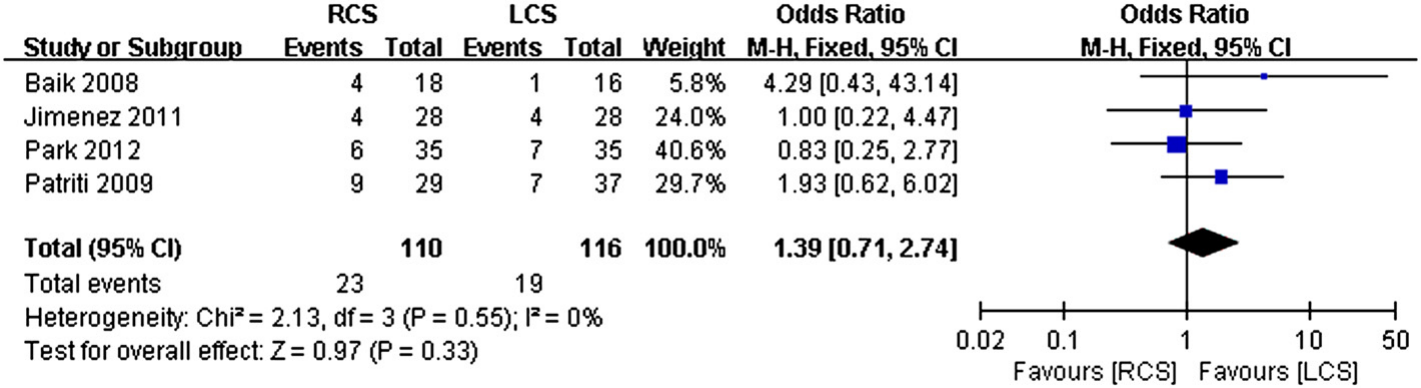
**Meta-analysis of the complication rates shown as a forest plot between robotic-assisted colorectal surgery (RCS) and laparoscopic colorectal surgery (LCS).** The odds ratios with 95% CIs are shown.

The complication rates were similar across studies, and there was no significant heterogeneity (*I*^2^ = 0). A meta-analysis of all of the studies in this index revealed that the complication rates of the two group were not obviously different (odds ratio (OR) 1.39, 95% CI 0.71 to 2.74, *P* = 0.33) (Figure 9).

### Meta-analysis of the pathological details

**Figure 10:**
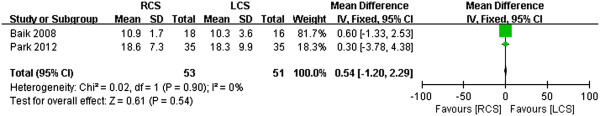
**Meta-analysis of the proximal margins shown as a forest plot between robotic-assisted colorectal surgery (RCS) and laparoscopic colorectal surgery (LCS).** The mean differences with the 95% CIs are shown.

**Figure 11:**
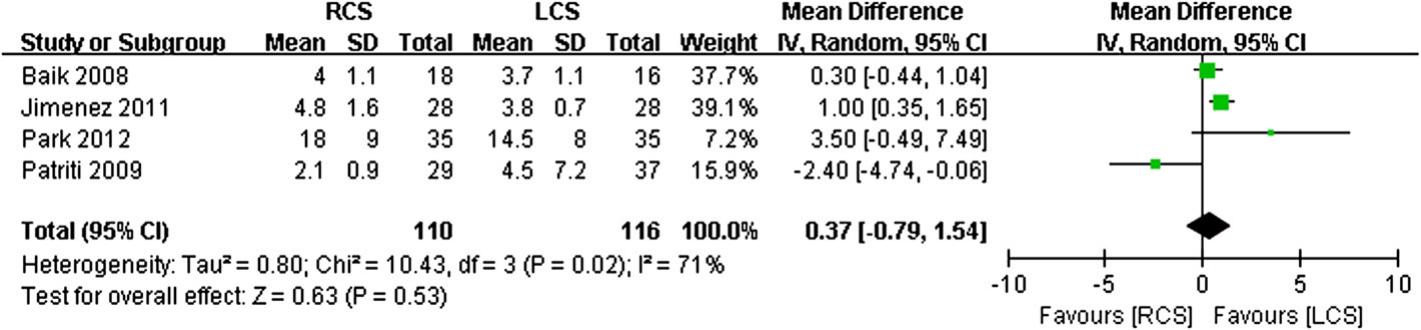
**Meta-analysis of the distal margins shown as a forest plot between robotic-assisted colorectal surgery (RCS) and laparoscopic colorectal surgery (LCS).** The mean differences with the 95% CIs are shown.

**Figure 12:**
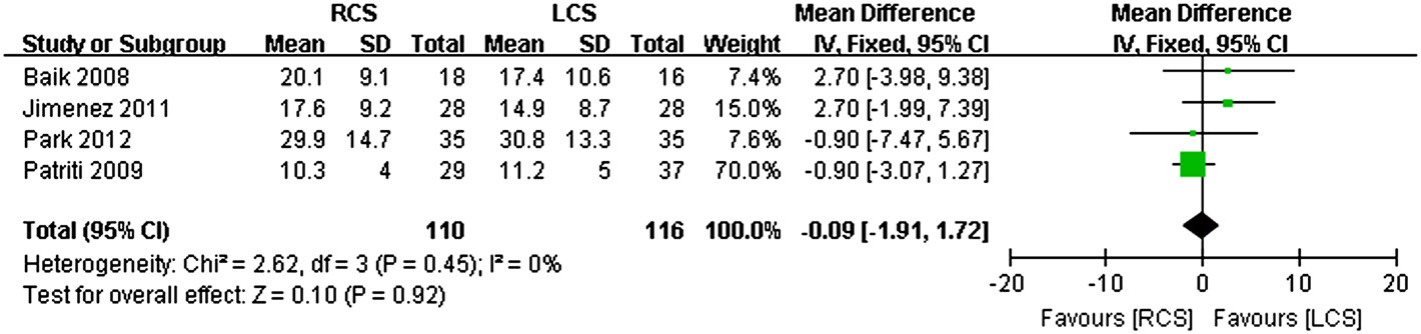
**Meta-analysis of the harvested lymph nodes shown as a forest plot between robotic-assisted colorectal surgery (RCS) and laparoscopic colorectal surgery (LCS).** The mean differences with the 95% CIs are shown.

Baik *et al*. [15] and Park *et al*. [13] reported proximal margin indices, and there was no difference in this regard (WMD -0.54, 95% CI -1.20 to 2.29; *P* = 0.54, *I*^2^ = 0) (Figure 10). All of the studies reported distal margins, and there was no difference between the two groups in this regard (WMD 0.37, 95% CI - 0.79 to 1.54; *P* = 0.53) (Figure 11). The studies exhibited significant heterogeneity (*I*^2^ = 71%); one study by Patriti *et al*. [14] found a high SD for the LCS group (7.2 cm), and, if this study was excluded, the heterogeneity was relatively low (*I*^2^ = 48%). The harvested lymph nodes were similar across all of the studies (*I*^2^ = 0), and analysis of the pooled data revealed that the two groups did not differ significantly in this regard (WMD 0.09, 95% CI -1.91 to 1.72; *P* = 0.92, *I*^2^ = 0) (Figure 12).

### Costs

As a newly developing surgical technique, it should prove to be safe and cost-effective until it is widely accepted worldwide. Thus, it is essential to evaluate the cost-effectiveness between RCS and LCS. In our meta- analysis, only one study addressed to this issue. Park *et al*. [13] reported that the overall hospital costs were significantly higher in the RCS group (US $12235 versus $10319.7), as showed in Table 2. Based on the limited data, it would be rash to make a conclusion that RCS is not cost-effective.

### Publication bias

**Figure 13:**
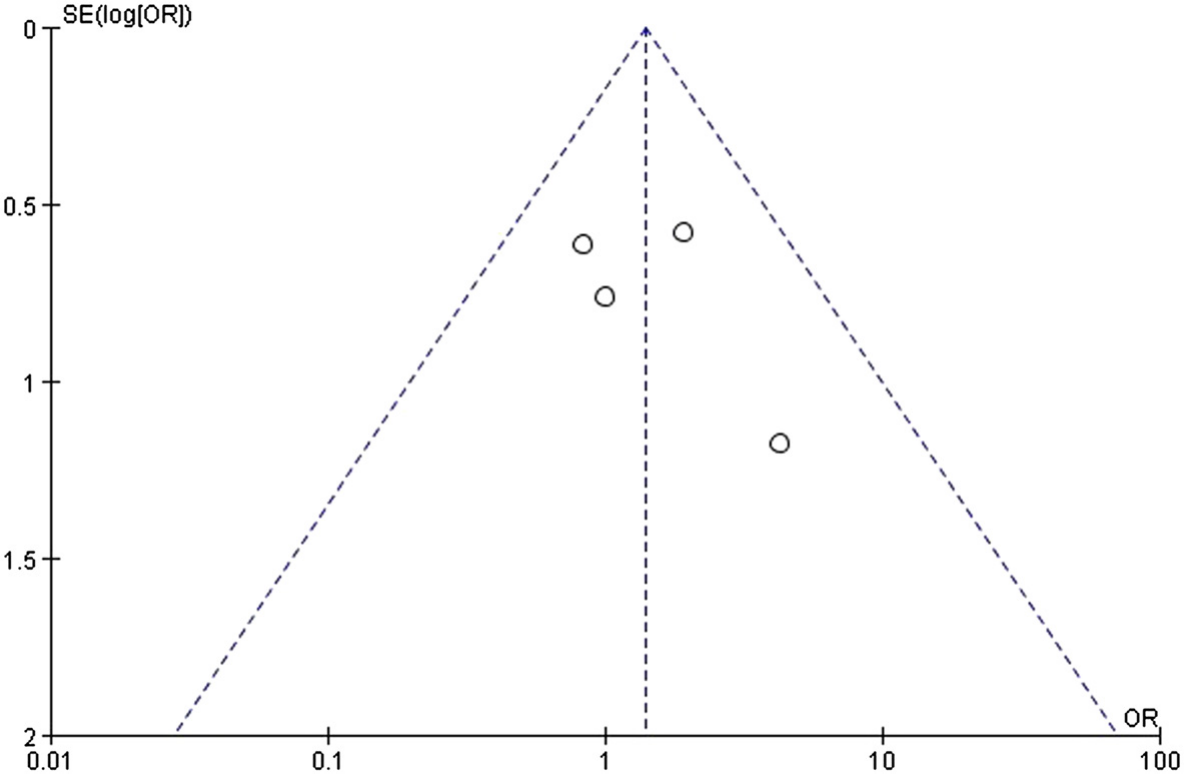
**Funnel plot used to assess a fixed-effects model of the results of all of the selected studies regarding the complications of robotic-assisted colorectal surgery (RCS) and laparoscopic colorectal surgery (LCS).** SE, standard error.

Complications were assessed by a funnel plot of the standard errors of the fixed-effect sizes between the RCS and LCS. This analysis revealed no evidence of publication bias or heterogeneity among the studies (*P* = 0.55, Figure 13).

### Sensitivity analysis

A sensitivity analysis was performed by excluding the low-quality studies (that is, those with high risk of bias). As shown in Figure 2, we considered the studies of Patriti *et al*. [14] to have high risks of bias. All variables were included for the sensitivity analysis. If one index was not enough available in a sufficient number of studies (that is, at least two), this index was excluded from further sensitivity analysis. The sensitivity analysis revealed that the operation times of the RCS group were longer than that of the LCS group (WMD 35.97, 95% CI 3.83 to 68.10, *P* = 0.03). There was no significant difference in the conversion rates of the two techniques (OR 0.51, 95% CI 0.10 to 2.49; *P* = 0.41). Analyses of the times to recovery of bowel function, complication rates, proximal margins, distal margins, and the harvested lymph nodes yielded similar results. However, the LOS of the RCS group were significantly shorter than those of the LCS group (WMD -1.35, 95% CI -2.34 to -0.37, *P* = 0.007).

## Discussion

Laparoscopic surgery has been applied in many surgical fields and has not only been shown to be effective and safe but also to have certain benefits over traditional open surgery [17-19]. However, limitations to the performance of laparoscopic surgery remain, including the high conversion rate to open surgery [20]. The advent of robot-assisted surgery has overcome some of the limitations of laparoscopic surgery and has been successfully applied to urology, general surgery, gynecology and other surgical fields [21,22]. Several meta-analyses have suggested that robotic laparoscopic surgery is feasible, produces similar perioperative outcomes and is oncologically safe [23,24]. Robotic surgery may result in shorter hospital stays, reduced blood losses and lower conversion rates, but it also may require increased operation time costs [24].

A previous meta-analysis [25] produced a similar conclusion regarding the comparison of robotic laparoscopic surgery to laparoscopic surgery in colorectal disease. However, the studies that were included in this previous meta-analysis were of relatively low quality, and the number of patients was relatively small. Additionally, the benefits of robotic surgery for colorectal disease remain controversial. Thus, we conducted this meta-analysis based on four RCTs to draw a clearer conclusion.

The present study is the first meta-analysis to compare robot-assisted and laparoscopic surgery for colorectal cancer based only on RCTs. The results of this meta-analysis revealed that RCS may provide additional benefits over LCS and that it is safe and effective [3,4,26]. Regarding operation times, RCS exhibited a tendency to take longer than LCS, but this difference was not significant (*P* = 0.06). This result is similar to that of a previous study [27]. In contrast to this view, other studies have reported that RCS requires more time than does LCS [28,29]. As the surgeons’ robotic experience increases and the techniques are improved, the operation times will be reduced [26]. However, in this regard, obvious heterogeneity existed among the studies; the reasons for this heterogeneity were as follows. First, the surgeries were performed by different surgeons who were not at the same point on the learning curve and had different amounts of experience. Additionally, the RCS group of one study contained a large number of patients who had previously undergone abdominal surgery [14], and it is well-known that previous surgery inevitably increases the difficulty of performing surgery.

EBL ranges between 90 ml and 320 ml for LCS [30] and between 20 ml and 486 ml for RCS according to a recently published review [31]. Our studies showed that the average EBL was 81.83 ml for RCS and 92.88 ml for LCS. Both studies reported no significant difference between the two groups in this regard. However, the pooled data revealed that the EBL was significantly lower for the RCS group than for the LCS group. This finding is consistent with those of previous studies [31]. However, the surgery that was performed by skilled surgical teams exhibited similarly low levels of EBL for the two techniques [4]. Due to lower EBL, it is possible to suggest that RCS may significantly reduce the probability of transfusion and might prevent the recurrence of cancer. A review reported that patients who receive more perioperative transfused blood are at greater risk for cancer recurrence and that this risk is independent of tumor stage at surgery [32]. It is well-known that the prognosis for colorectal cancer after surgery is highly dependent on the cancer stage at surgery. However, the EBL during colorectal cancer surgery influences long-term survival according to a previous study [33]. Massive blood loss during colorectal cancer surgery is probably related to advanced disease or the recurrence of cancer [34], preoperative therapy (neoadjuvant therapy with either chemoradiation or chemotherapy alone), unclear anatomy [35] and the lack of experience of the surgical team rather than the type of surgical approach. Because only two studies reported EBL in this analysis, further studies are necessary to prove whether RCS results in lower blood loss.

We found that the conversion rate was significantly lower in the RCS group and there was no significant heterogeneity across the four studies. Thus, the reduction in conversion rate may be one of the key benefits of RCS. Conversion rate is a valuable index of surgical quality. Lower conversion rates are associated with fewer postoperative complications [20]. The reduction in conversion rate may be attributable to the fact that robotic surgery can facilitate certain steps in colorectal procedures. These procedures include splenic flexure takedown, dissection of the inferior mesenteric vessels, dissection in the narrow pelvis [36], et cetera.

As observed in the studies, robotic surgery has other advantages. The time to the recovery of bowel function was shorter in the RCS than in the LCS group. This reduction may be another potential benefit of RCS because faster returns to normal diets are associated with faster recoveries. We discovered that the LOS was not significantly different between the two groups; indeed, LOS was similar between the two techniques [26]. The complication rates of the patients who underwent RCS and LCS were similar. This finding also demonstrates that RCS is as safe and feasible as LCS [37]. Regarding the pathological details, there were no differences between the two techniques in terms of proximal margins, distal margins or harvested lymph nodes.

Although we conducted this meta-analysis based on four RCTs, this meta-analysis still has the following limitations. First, the included studies were not double-blind studies or even single-blind studies; the studies may have been biased; therefore, the interpretation of their results may also have been biased. Second, although we focused on colorectal surgery, different types of diseases (for example, rectal cancer, sigmoid colon cancer, right colon cancer) that involved different types of surgery were included, which may have caused heterogeneity. Third, the surgery in the included cases were not performed by the same surgeon, and the experience and technique of the surgeons may have affected some of the indices, such as the operation time, EBL and conversion rate. However, in this meta-analysis, no significant heterogeneities among the four studies were found for conversion rate or EBL; only operation time exhibited significant heterogeneity. Fourth, long-term follow-up evaluations assessing oncologic outcomes were not available from the selected studies, and only Patriti *et al*. [14] reported medium-term outcomes. Fifth, the available included RCT studies were limited, and the numbers of operations were small; however, other RCT studies comparing RCS and LCS, such as the ACOSOG Z6051 and ROLARR studies, are ongoing [38]. Finally, only one study compared costs between RCS and LCS [13]. This study revealed that the overall hospital costs were significantly higher for RCS than for LCS. Robotics is a new trend in colorectal surgery, and more attention has been paid to the high cost that is associated with this new technique. A variety of studies have reported that the cost of robotic colorectal surgery is higher than that of laparoscopic surgery [3,39,40]. The high capital and running costs of robotic systems have precluded their widespread use in many countries [41]. Furthermore, one study reported that RCS does not provide sufficient advantages over LCS but significantly increases the total cost [38]. Future studies should assess the cost-effectiveness of RCS based on long-term oncologic outcomes and functional results.

## Conclusions

This meta-analysis suggests that RCS is safe and effective and has some advantages over LCS. These benefits include a reduced conversion rate, reduced blood loss and reduced time to recovery of bowel function. Regarding the other variables, such as operation time, complication rate and LOS, there were no significant differences between the two groups. However, RCS was associated with a significant increase in total costs relative to LCS. Further well-designed, prospective controlled randomized trials should be conducted to assess the financial benefits and the long-term oncologic outcomes of RCS.

## Abbreviations

BMI: body mass index; EBL: estimated blood loss; LCS: laparoscopic colorectal surgery; OR: odds ratio; RCS: robotic-assisted colorectal surgery; RCT: randomized controlled trial; WMD: weighted mean difference.

## Competing interests

The authors declare that they have no competing interests.

## Authors’ contributions

GXL, ZHZ, YWY and SHL conceived the study and participated in its design, the literature research, data extraction, manuscript drafting. RL, JRC, SSD and HJD participated in manuscript drafting, and editing. All authors read and approved the final manuscript.
